# Fear-avoidance beliefs are associated with a high fat content in the erector spinae: a 1.5 tesla magnetic resonance imaging study

**DOI:** 10.1186/s12998-019-0234-2

**Published:** 2019-03-15

**Authors:** Eddo Wesselink, Edwin de Raaij, Philip Pevenage, Nick van der Kaay, Jan Pool

**Affiliations:** 1 Research Group Lifestyle and Health, University of Applied Sciences Utrecht, Heidelberglaan 7, 3584 CS Utrecht, The Netherlands; 2 Department of Health Sciences, VU University, Amsterdam, The Netherlands; 3 the EMGO Institute for Health and Care Research, Amsterdam, The Netherlands; 4 MRI Centre, Amsterdam, The Netherlands; 5 Paramedical Centre Fytac, Genemuiden, The Netherlands; 6 Physiotherapy van der Kaay, Leiden, The Netherlands

**Keywords:** Adipose tissue, Chronic low back pain, Avoidance behavior, MRI

## Abstract

**Background:**

Intramuscular adipose tissue (IMAT) is a feature of degenerative muscle composition and is a common feature in populations with chronic low back pain (CLBP). Avoidance behavior is a possible cause of morphological muscle composition due to disuse of the paraspinal muscles. Therefore it is of clinical interest to determine the association between fear-avoidance beliefs and IMAT of the paraspinal muscles in populations with CLBP.

**Methods:**

In this cross-sectional study, we examined twenty-four adults, featuring a mean age of 48.63 years (SD ± 14.73), with CLBP. Axial T2-weighted Magnetic Resonance Imaging (MRI) images were selected on the same level as the intervertebral disc of segments L4-L5 and L5-S1. After determine the region of interest, the amount of IMAT was measured by an automatic-threshold method to distinguish fat from muscle tissue. Fear-avoidance beliefs were measured with the Fear-Avoidance Beliefs Questionnaire, with regard to Physical Activity (FABQ-PA). Bivariate correlation and multiple regression analysis were used to determine the association between IMAT of the paraspinal muscles and fear-avoidance beliefs.

**Results:**

There is a significant bivariate association between the FABQ-PA and ES IMAT (*r* = 0.484, *P* = 0.017), but not for LMM (*r* = 0.228, *P* = 0.284). The association between the FABQ-PA and ES IMAT remained moderate after adjusting for covariates (β = 0.381, *P* = 0.028).

**Conclusion:**

Fear-avoidance beliefs are moderately associated with ES IMAT and poorly associated with LMM IMAT in a population with CLBP. Results should be interpreted with caution due to a small and selected study population.

## Background

Low back pain (LBP) is a common musculoskeletal condition that affects up to 85% of the general population during their lives [1], with a high recurrence risk of 44 to 78% [2]. Chronic low back pain (CLBP) is considered to be a multifactorial condition due to a constant interaction between physical (levels of conditioning and loading exposures), psychological (stress, cognition and emotion) and social systems (culture, work, home and environment) [3].

One of the anatomical biomarkers, as part of the physical system in people with CLBP is the presence of a degenerative muscle composition in the paraspinal muscles [4–8]. Degenerative muscle composition is characterized by a decrease in the cross-sectional area (CSA) of the muscle [4, 5], and an increase of fatty infiltration, also known as “intramuscular adipose tissue” (IMAT) [6–8]. The burden of proof for the CSA as a surrogate for degenerative muscle composition is inconclusive and contradictory. In some studies, a decrease in the CSA of the paraspinal muscles is associated with LBP [9, 10], but other studies disagree [11–13]. Shahidi et al. [14] show that paraspinal muscle tissue changes are more complex than atrophy alone. IMAT appears to be a better representation of degenerative muscle composition and is a feature of decreased muscle structure and quality. In addition to IMAT, a slow-to-fast muscle fiber transformation [11], high levels of inflammation and decreased vascularity [14] are also described as features of structural remodeling of degenerative muscle tissue.

Several studies have shown an association between IMAT of the paraspinal muscles and CLBP [6]. For example, more IMAT was found in the lumbar multifidus (LMM) of patients with CLBP (23.6%), compared to people without complaints (14.5%) [15]. In addition, significantly more IMAT was found in the paraspinal muscles of patients with CLBP compared to acute LBP [16].

One of the possible explanations for the accumulation of IMAT in people with CLBP is altered individual-specific motor behaviors of paraspinal muscles [17]. It is known that IMAT is associated with decreased muscular metabolic activity [18, 19], which can lead to a reduction of intramuscular mitochondrial oxygenation of glucose, thus increasing insulin resistance and intramuscular triacylglycerol levels [20]. Intramuscular triacylglycerol levels can lead to alternative communication (adipose muscle crosstalk) between satellite cells and macrophages, where myogenic adult stem cells differentiate to fat cells instead of myocells [21], with negative consequences for the development of new sarcomeres [22, 23], thus leading to a degenerative muscle composition.

Avoidance behavior is a highly associated with pain-related factors contributing to muscle inhibition [24], and altered individual-specific motor behaviors of paraspinal muscles [17], because people with negative cognitions about pain can be afraid to move when a pain experience is considered to be dangerous [25, 26]. These pain-related factors are highly associated with a poor treatment outcome in patients with CLBP [27].

From this clinical perspective, an association between avoidance behavior and IMAT is of clinical interest because it could bear consequences for the choice of treatment and exercise intensity in people with CLBP. For example, low-intensity exercises like motor control rehabilitation presumably have a low impact on paraspinal metabolic activity and muscle quality [28, 29], when pain-related avoidance beliefs are present. It has however not yet been investigated whether avoidance behavior is associated with IMAT in a population with CLBP. Hence the research question is: What is the association between fear-avoidance beliefs and IMAT of the paraspinal muscles in patients with CLBP?

## Methods

This cross-sectional study was conducted in an independent and specialized centre for Magnetic Resonance Imaging (MRI) diagnostics. MRI images were generated from participants who were referred for medical diagnostic research due to their CLBP and were blinded to MRI-findings when completing the questionnaires. The participants did not perform any additional actions for this study and consented to the use of anonymized personal information through informed consent. The Institutional Review Board (department of health studies) of HU University of Applied Sciences Utrecht approved the study protocol, reference number: 35_010_2016.

### Participants

Patients met the inclusion criteria if they were 18 years or older and had experienced (chronic) LBP lasting longer than 12 weeks. If there was a history of lumbar surgery, neurological disorders, spinal deformities, and recent traumatic incidents, patients were excluded from participation. MRI images with fat suppression were considered unsuitable and are excluded from this study.

### Procedure imaging

Low-field Tesla 1.5 MRI images (GE Medical Systems, USA, Siemens Healthcare, Erlangen, Germany) are used with a repetition time of 4110–4400 milliseconds (ms) and an echo time of 132 ms. The slice thickness was 3 mm (mm) and the field of view 200 × 168.6 mm. The patients were placed supine with their hips and knees slightly bent (30 degrees) to maintain a neutral position of the lumbar vertebral column. The transverse recordings were selected at the intervertebral disc of L4-L5 and L5-S1 using T2-weighted sagittal images of the lumbar spine.

### IMAT

**Fig. 1:**
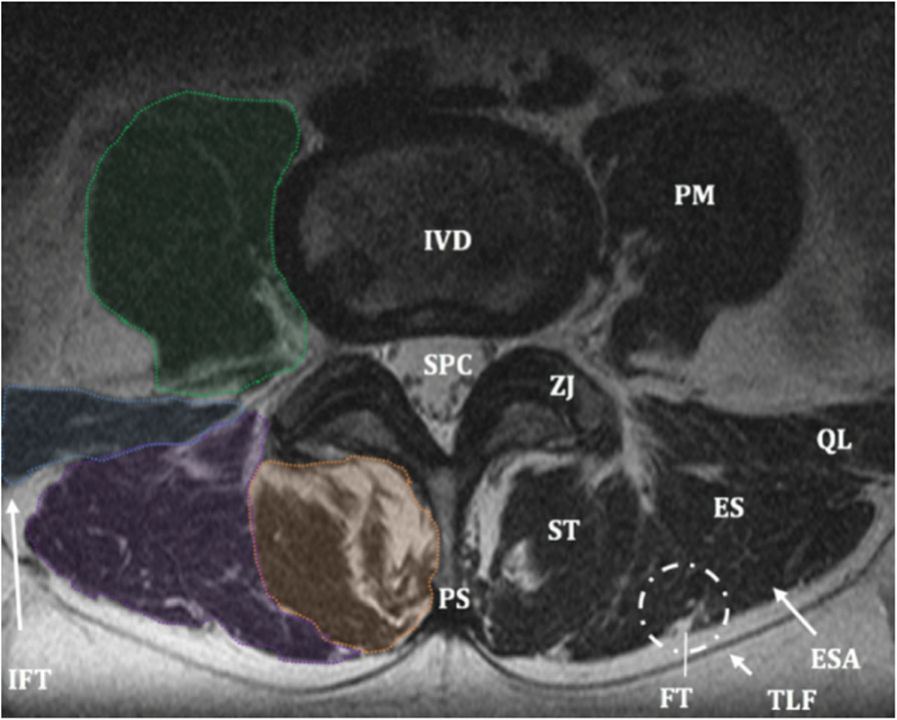
Anatomical cross-references used in this study. Abbreviations: *IVD* intervertebral disc, *FT* fatty tent, *ES* erector spinae, *ST* spinotransversal muscles (lumbar multifidus), *QL* quadratus lumborum, *PM* psoas major, *SPC* spinal canal, *ZJ* zygapophyseal joint, *TLF* fascia thoracolumbalis, *IFT* intrafascial triangle, ESA, aponeurosis of the ES. Orange polygonal circumference measurement = total region of interest lumbar multifidus; purple polygonal circumference measurement = total region of interest erector spinae, blue polygonal circumference measurement = quadratus lumborum, green polygonal circumference measurement = psoas major

**Fig. 2:**
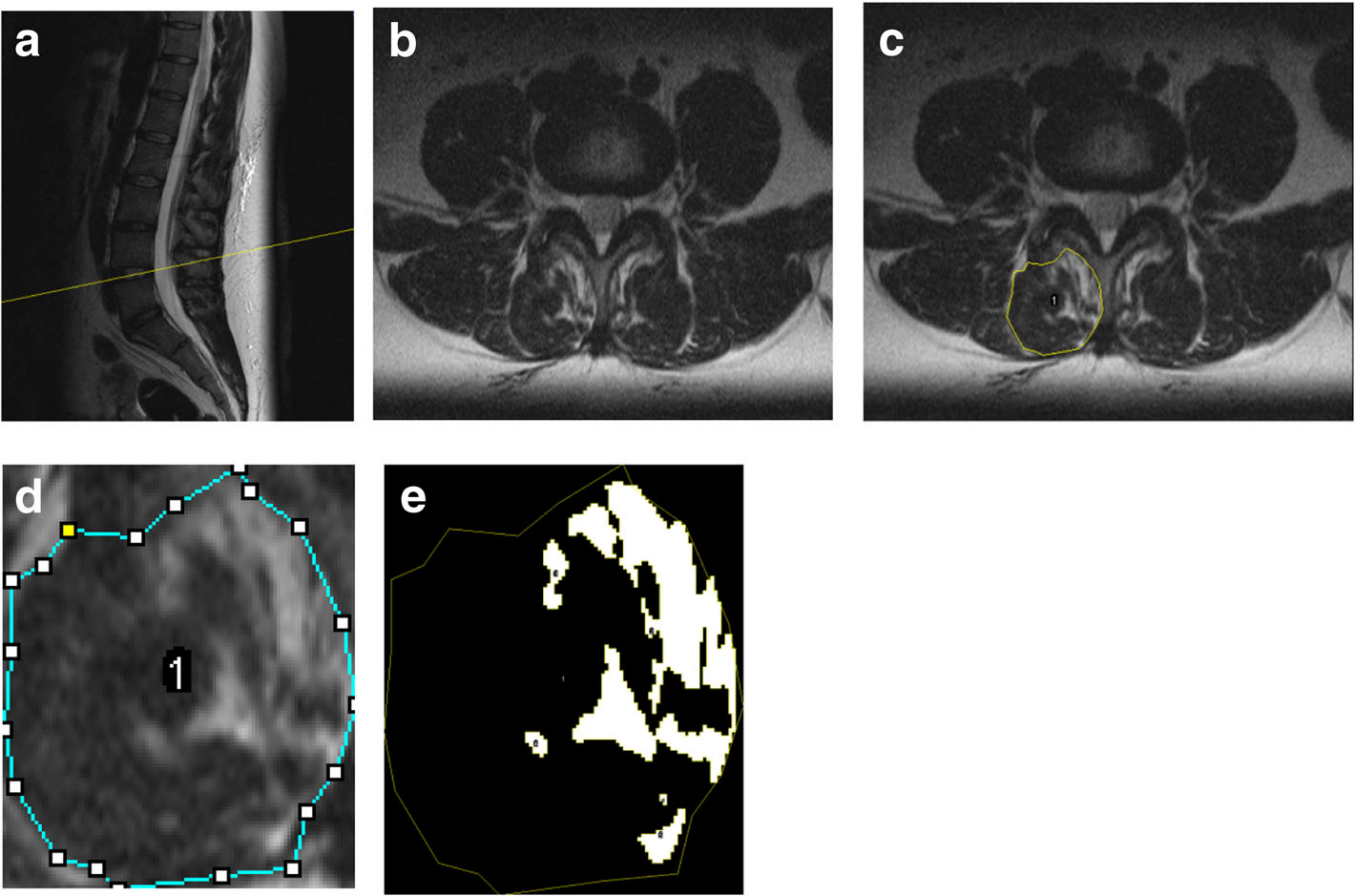
Sagittal T2-weighted images (2**a**), with a selection of transverse to L4-L5 intervertebral disc, (2**b**), with a polygonal circumference measurement of the lumbar multifidus (2**c**), cropping image (2**d**) and automatic segmentation (2**e**)

MRI data was analyzed using ImageJ 1.50i (Java-based version, public domain NIH Image Software; Research Services Branch). The T2-weighted transversal images were converted to an 8-bit gray scale. The CSA of the Erector Spinae (ES) and the LMM were bilaterally outlined at each level and were defined as the TROI. Anatomical cross-references of the LMM and ES muscles are used as proposed by Crawford et al. [30] (Fig. 1). Fatty substances between the aponeurosis of the ES and the posterior layer of the fascia thoracolumbalis, the ‘fatty tent’ between the dorsal aponeurosis of the iliocostalis and longissimus, and intrafascial triangles were not included and are considered as extramuscular fat [30]. After cropping the region of interest, the amount of IMAT was measured by an automatic-threshold method to distinguish fat from muscle tissue using a histogram (Fig. 2) [31]. The extent of IMAT was calculated by dividing the Functional Region of Interest (FROI) relative to the Total Region of Interest (TROI) by the following formula: %IMAT = FROI / TROI × 100%. This methodology is widely accepted, and features high intra-rating reliability (ICC > 0.75) [32], and concurrent validity with phantom images [33].

### Pain-related fear-avoidance beliefs

Levels of fear-avoidance beliefs were determined by the Fear-Avoidance Beliefs Questionnaire (FABQ). The FABQ consisted of 16 questions where the answers varied between 0 (disagree) to 6 (fully agree). Only the subdomain Physical Activity (FABQ-PA), the total sum ranging from 0 to 24 points, has been used in this study. The total score is the sum of items 2 to 5, with a higher score representing a greater degree of pain-related fear-avoidance beliefs [34]. A score of 14 points or higher indicates the presence of pain-related fear-avoidance beliefs. The FABQ-PA has a good inter-rater reliability of ICC = 0.90 [34], and an adequate concurrent validity with the Tampa Scale for Kinesiophobia, *r* = 0.62 in people with CLBP [34].

### Potential confounders

A strong association has been found between IMAT and disc degeneration [35–37], age [38–40], extent of physical activity [36], degree of pain [7], and gender [41] and have been included in this research as potential confounders.

### Self-reported questionnaires

Pain intensity was measured using a numeric pain-rating scale (NPRS). The participants assessed their average pain for the week preceding the MRI on a scale of 0 (no pain) to 10 (most severe pain). The NPRS has been proven as a valid and reliable measurement tool for measuring pain intensity [42]. Physical activity was measured by asking the participants how many hours a week they participated in sporting activities on average, for the four weeks prior to the MRI.

### Disc degeneration

Intervertebral degenerative changes of the disc were determined by T2-weighted sagittal images of the lumbar spine, as they provide a comprehensive impression of the disc structure [43]. The modified Pfirrmann classification of Griffith et al. [44] was used to determine the degree of disc degeneration, ranging from 1 (no degeneration) to 8 (final stage of degeneration). This research method has been proven as reliable [44].

### Statistical analysis

A statistical analysis was performed using IBM SPSS Statistics 20.0. Descriptive statistics were tested together with the Shapiro-Wilk test to determine the distribution of data. The average difference in IMAT between the LMM and the ES was tested by the paired samples t-test. A bivariate correlation matrix was used to assess the independent relationship between IMAT and FABQ-PA, as well as the potential covariates. A Pearson correlation coefficient (*r)* was used when the bivariate association met the assumption of the parametric test [45]. A non-parametric test (Spearman’s Rho) was used when the bivariate association did not meet the assumptions of the parametric test. The bivariate association between gender and IMAT was tested by a point biserial coefficient (r_pb_).

A multiple regression analysis was used to determine if IMAT associated with FABQ-PA after correcting for the variance due to the covariates. At first, IMAT and FABQ-PA were entered in the regression model. Next, covariates with a significant relationship (*P* < 0.10) with IMAT were all entered simultaneously in the second step by a enter method. The standardized coefficients (βeta) were calculated for the association between IMAT (LMM and ES separately) and the FABQ-PA after they were corrected for covariates and are considered significant at *P* < 0.05. An analysis of variance (ANOVA) was used to determine whether the regression model explained a significant proportion of the statistical variance.

## Results

### Demographic characteristics

Demographic characteristics of the sample are shown in Table 1. All descriptive statistics showed a normal distribution of data (Shapiro-Wilk *P* > 0.05). Twenty-four patients met the inclusion criteria, with a mean age of 48.63 years (SD ± 14.73). The average score on the FABQ-PA was 14.92 (SD ± 5.39), of which 70.8% scored higher than 14 points. LMM IMAT (22.03%, SD ± 11.88) was significantly higher (*P* = 0.046) than ES IMAT (17.72%, SD ± 11.66), with an average difference of 4.28%.

**Table 1:** Demographic and clinical characteristics of participants

Variable	Value
Age (SD)	48.63 (14.73)
Females (%)	13 (52)
Physical Activity (weekly)	
0–2 h (%)	8 (32%)
2–5 h (%)	6 (24%)
5–10 h (%)	7 (28%)
> 10 h (%)	3 (12%)
NPRS (SD)	5.58 (1.91)
FABQ-PA (SD)	14.92 (5.39)
LMM IMAT (SD)	22.03% (11.88)
ES IMAT (SD)	17.72% (11.66)

Abbreviations: *FABQ-PA* Fear-Avoidance Beliefs Questionnaire subdomain Physical Activity, *LMM* Lumbar Multifidus, *ES* Erector Spinae, *IMAT* Intramuscular Adipose Tissue, *NPRS* Numeric Pain Rating Scale, *SD* standard deviation

NOTE. Values are presented as mean ± SD or %. *N* = 24

### Bivariate correlation analysis

Table 2 shows the bivariate correlation matrix. Data met the full assumption for parametric testing. There was a significant association between FABQ-PA and ES IMAT (*r* = 0.484, *P* = 0.017), but not the LMM (*r* = 0.228, *p* = 0.284). LMM IMAT was found to be significantly associated with the age (*r* = 0.678, *p* < 0.001), the extent of disc degeneration on L4-L5 (*r* = 0.515, *P* = 0.010) and L5-S1 (*r* = 0.477, *P* = 0.018). LMM IMAT was not significantly associated with, gender, physical activity and pain intensity (*P* > 0.1). ES IMAT also showed a significant relationship with age (*r* = 0.472, *p* = 0.020), disc degeneration of L4-L5 (*r* = 0.480, *p* = 0.018) and approximate statistical significance for disc degeneration on L5-S1 (*r* = 0.336, *p* = 0.109) and physical activity (*r* = − 0.350, *p* = 0.094). No significant association has been found for ES IMAT with gender and pain intensity (*P* > 0.1).

**Table 2:** Bivariate correlations between the lumbar IMAT infiltration and gender, age, physical activity, NPRS mean, disc degeneration and fear-avoidance beliefs

		LMM IMAT	ES IMAT
Gender	*r*_pb_	−0.103	0.088
	p	0.630	0.683
Age	*r*	0.678	0.472
	p	< 0.001	0.020
Physical activity	*r*	−0.205	−0.350
	p	0.337	0.094
NPRS mean	*r*	0.075	−0.188
	p	0.728	0.379
Disc degeneration L4-L5	*r*	0.515	0.480
	p	0.010	0.018
Disc degeneration L5-S1	*r*	0.477	0.336
	p	0.018	0.109
FABQ-PA	*r*	0.228	0.484
	p	0.284	0.017

Abbreviations: *NPRS* Numeric Pain Rating Scale, *FABQ-PA* Fear-Avoidance Beliefs Questionnaire subdomain Physical Activity, *IMAT* intramuscular adipose tissue, *LMM* Lumbar Multifidus, *ES* Erector Spinae

*r*_pb_ = Point biserial coefficient

### Multiple regression analysis

Tables 3 and 4 shows the multiple regression analysis. The significant explained variance of ES IMAT, physical activity, age and disc degeneration of L4-L5 (model 2) was 55.5% (ANOVA *P* = 0.003). After adjusting for confounders, the association between the FABQ-PA and ES IMAT was moderate (β 0.381, *P* = 0.028). The significant explained variance of LMM IMAT, disc degeneration and age (model 2) was 54.5% (ANOVA P = 0.003). The association between the FABQ-PA and LMM IMAT was poor (β 0.168, *P* = 0.307) after adjusting for confounders.

**Table 3:** Multiple regression analysis IMAT ES L4-L5 & L5-S1

	Variable	Unstandardized coefficients			Standardized coefficients	*P*-value
		B	95% CI		βeta	
			Lower	Upper		
M1	(Constant) FABQ-PA	3.526 0.952	−8.655 0.191	15.707 1.713	0.484	0.554 0.017
M2	(Constant) FABQ-PA DD L4 Age PA	−9.985 0.750 0.679 0.354 −3.355	−26.748 0.092 −1.817 0.040 −7.126	6.778 1.409 3.175 0.668 0.416	0.381 0.110 0.447 −0.305	0.228 0.028 0.576 0.029 0.078

Abbreviations; *M* Model, *IMAT* Intramuscular Adipose Tissue, *PA* Physical Activity, *DD* Disc Degeneration, *FABQ-PA* Fear-avoidance beliefs questionnaire subdomain physical activity, *ES* Erector Spinae

**Table 4:** Multiple regression analysis IMAT LMM L4-L5 & L5-S1

	Variable	Unstandardized coefficients			Standardized coefficients	*P*-value
		B	95% CI		βeta	
			Lower	Upper		
M1	(Constant) FABQ-PA	15.196 0.456	1.394 −0.406	28.998 1.319	0.228	0.032 0.284
M2	(Constant) FABQ-P Age DD L4 DD L5	−13.341 0.336 0.430 0.766 1.003	−30.157 −0.334 0.104 − 1.777 − 1.333	3.475 1.006 0.756 3.309 3.140	0.168 0.533 0.122 0.174	0.362 0.307 0.012 0.536 0.338

Abbreviations; *M* Model, *IMAT* Intramuscular Adipose Tissue, *DD* Disc Degeneration, *FABQ-PA* Fear-avoidance beliefs questionnaire subdomain physical activity, *LMM* Lumbar Multifidus

## Discussion

This study has shown a moderate relationship between pain-related fear-avoidance beliefs and ES IMAT. Avoidance behavior can be seen as a common-sense response to dealing with LBP when people have negative thoughts about their pain [24, 46], which are associated with altered motor patterns of paraspinal muscles [17, 47] and reduced physical activity [48]. One added value of our results could be its utility as additional evidence showing why avoidance behavior is not a recommended approach in CLBP.

A small association has been found between fear-avoidance beliefs and LMM IMAT, in contrast with ES IMAT. This difference may be due to different substitution patterns of the LMM as compared to other spinal muscles, which are caused by a long-loop inhibition through the medial ramus of the dorsal radix and are probably more related to pain-induced vertebral pathology [49]. Shahidi et al. [39] noted that the lumbar multifidus degenerates in people with chronic degenerative lumbar spine pathology. This corresponds with our findings, where LMM IMAT is highly associated with age (*r* = 0.678, *P* < 0.001*)* and disc degeneration (L4-L5 *r* = 0.515, *P* = 0.010 and L5-S1 *r* = 0.477, *P* = 0.018). In addition, rapid atrophy of the LMM has been noted within 3 days after intervertebral lesions in a porcine model [50], or pain onset in humans [9]. After pain onset, pain-induced reflex inhibitory mechanisms and disturbance in coordination are also noted in the LMM [51]. Nevertheless, we know that muscle tissue changes are more complex than atrophy alone in people with CLBP [13], and therefore a clear explanation for the underlying mechanisms of different outcomes in ES IMAT and LMM IMAT remains hypothetical.

Degenerative muscle composition with a high fat content increases with age, appears to progress faster in the LMM (0.24% per year) than the ES (0.13% per year) and corresponds to findings from this study [38]. The standardized coefficient (βeta) in the final model of the multiple regression analysis was higher for LMM IMAT (β = 0.533) and less so for ES IMAT (β = 0.447).

People with CLBP demonstrate altered individual-specific motor (control) behaviors [17, 47] and show discrete loss of cortical organization of inputs to paraspinal muscles which could lead to differential central activation [52, 53]. In this case, motor rehabilitation is suggested as a possible effective treatment to restore optimal control in LBP [54]. To restore full motor control of the lower back, it is doubtful whether low-intensity exercises like motor control rehabilitation alone is effective to improve paraspinal muscle function when high levels of intramuscular fat are present. We think that ES IMAT could be an adverse secondary consequence of avoidance behavior due to altered individual specific motor patterns [17, 55], with reduced muscular metabolic activity [18, 19]. From this perspective, cognition-targeted motor control training combined with pain neuroscience education could be a more appropriate intervention to improve muscle function, also because it has been proven more effective than current best-evidence physiotherapy for improving physical function and pain cognitions in people with CLBP [56]. However, in our study it cannot be determined whether the association of fatty infiltration of the paraspinal muscles and pain-related fear-avoidance beliefs is the cause or effect of one another. To demonstrate a causal relationship between pain-related fear-avoidance beliefs and IMAT of the paraspinal muscles, studies with a longitudinal design are recommended.

This study shows that the average LMM IMAT was 22.03%, and ES IMAT 17.72%. Other studies took diverse IMAT levels for both the LMM and ES [6, 57]. Besides population-based differences like age or levels of disability, such controversial results could be explained by differences in methodology (quantitative versus qualitative measurement methods, MRI versus computed tomography scan) [30, 31]. Despite discrepancies in methodology, histological studies demonstrated that IMAT observed by MRI strongly corresponds to histologic evidence of paraspinal morphology and is therefore considered a valid method for quantifying the degree of fatty infiltration [31]. Some studies have proven reliable at distinguishing fat from muscle tissue in conventional T1-weighted MRI images based on pixel intensity and histographic methodology [58, 59]. In order to compare different studies in future investigations, it is recommended to standardize threshold and segmentation procedures.

### Study limitations

A limitation in this study is the use of T2-weighted spin echoes, which can be more difficult to correct due to other changes that may occur in and around the muscle [59]. Maillard et al. [60] describes a rapid extension of the T2 relaxation time of (muscle) inflammation to as much as 100 ms/s, which closely matches the T2 relaxation time of fat (130–150 ms/s) and may affect the validity of the measurement. This difference is larger in T1-weighted spin echoes and is therefore recommended for subsequent research to quantify the degree of fatty infiltration in conventional MRI images. However, the concurrent validity and reliability are shown to be excellent in T2-weighted images [32]. Either way, MRI-based methods as DIXON/IDEAL [61] seems to be most accurate to distinguish fat from muscle tissue than normal T1- or T2-weighted spin echoes and are therefore widely recommended for subsequent testing [62, 63]. Since imaging research in this population was used for usual care, and not for the benefit of this study, it was not possible to apply these methods.

In this study, the average age was 48.63 years (SD ± 15.29) and 70.08% of the included participants were classified with pain-related fear-avoidance beliefs (FABQ-PA > 14). These factors may have led to selection bias, and for this reason it may be difficult to generalize our results to an elderly population or population with chronic lower back pain without pain-related fear-avoidance beliefs. Larger epidemiological studies of the general population can provide valuable research data. In this study, the multiple regression analysis contained one outcome variable and three covariates with 24 included participants. This model did not fully meet the recommended limit of a multiple regression analysis of at least ten observations per variable, which may have lowered the validity of the analysis [64]. An investigation using a larger population is recommended in the future.

## Conclusions

This study has shown a moderate association between pain-related fear-avoidance beliefs and IMAT in ES, but a poor association with LMM IMAT. The small and selected study sample may have lowered the validity of our results, and conclusions have to be interpreted with caution. More longitudinal research with a larger population is required to demonstrate a causal relationship. MRI-based methods like DIXON/IDEAL are recommended for further research.

## Abbreviations

ANOVA: Analysis of variance
CLBP: Chronic low back pain
CSA: Cross-sectional area
ES: Erector spinae
FABQ-PA: Fear-avoidance beliefs questionnaire physical activity
FROI: Functional region of interest
IMAT: Intramuscular adipose tissue
LBP: Low back pain
LMM: Lumbar multifidus
MRI: Magnetic resonance imaging
ms: Milliseconds
NPRS: Numeric pain rating scale
ROI: Region of interest
TROI: Total region of interest
